# Plant-Based Innovations for the Transition to Sustainability: A Bibliometric and in-Depth Content Analysis

**DOI:** 10.3390/foods11193137

**Published:** 2022-10-09

**Authors:** Małgorzata Krzywonos, Katarzyna Piwowar-Sulej

**Affiliations:** 1 Department of Process Management, Wrocław University of Economics and Business, Komandorska 118/120, 53-345 Wrocław, Poland; 2 Department of Labor, Capital and Innovation, Wrocław University of Economics and Business, Komandorska 118/120, 53-345 Wrocław, Poland

**Keywords:** plant-based innovation, food, sustainability, sustainable development, bibliometric analysis, content analysis

## Abstract

Plants maintain the ecological equilibrium of the earth and stabilize the ecosystem. Today, traditional commodities and new value-added markets can be served simultaneously. There is significant biosource and bioprocess innovation for biobased industrial products. Furthermore, plant-based innovation is associated with the transition to sustainability. This study performed a bibliometric and in-depth content analysis to review plant-based innovations in the research field between 1995 and 2022. A set of 313 articles was identified from the Scopus and Web of Science databases. Different analytical scientometric tools (topic mapping and overlay visualization networks) were used to analyze 124 articles; the most influential countries, institutions, authors, journals and articles were identified. Through in-depth studies, based on the grounded theory approach, five leading research areas related to plant-based innovation were determined: (1) agricultural/environmental innovation, (2) plant-based food or feed innovation, (3) innovation within the medical/pharmaceutical research area, (4) technology-related innovation and (5) economic/business aspects of plant-based innovations. Future research directions include exploring less examined and new topics, such as the sustainability implications of incorporating various plant-based foods and Industry 4.0 in plant-based innovation, and linking and developing findings from different research areas.

## 1. Introduction

Innovation is currently one of the main topics of debate in the literature devoted to economics [1] and management [2]. However, global challenges (urgent, complex and interconnected problems) and changes in the structure of knowledge production have led to diverse definitions and typologies of innovation [3].

In general, innovation is a process of formulating, applying, launching and developing a creative idea and guiding it as it matures and comes to fruition [4]. The first sociological theory of innovation was articulated by Tarde [5], who focused on explaining social changes (including economic-regime and industrial changes) and for whom innovation meant invention. However, Schumpeter is considered the precursor of the concept of innovation. He distinguished innovation from invention and identified innovation as the introduction of new products, applying new methods of production or sale, entering new markets, obtaining new sources of raw materials and implementing new industrial structures [6].

In Schumpeter’s theory, entrepreneurs play a crucial role in innovation and, as a result, in creating economic growth and development [7]. However, recent publications show innovation as an interactive process involving many actors [8]. Economic growth is mainly associated with technological innovation, defined as developing new products and production techniques [9]. This innovation leads to higher productivity and provides more goods and services that meet the needs of consumers [10,11,12]. However, technology can produce both benefits and harms.

Innovation generally does not occur in isolation [13]. As LeBel [14] states, “in a globalizing world in which a rising population places growing pressure on the stock of natural resources, sustainable growth depends more than ever on how innovation can be nurtured. Innovation is what may be considered as knowledge capital, and it stands in distinction to traditional measures of capital, notably physical stock” [14] (p. 335). The concept of responsible innovation is increasingly being studied, emphasizing social and ethical issues in innovation (not only profits related to innovation) [15,16,17]. Some producers offering innovative solutions to customers achieve an 80% minimum for the proportion of innovative products that meet sustainability-oriented criteria [18]. However, about two-thirds of such products remain in small market niches—a problem for the transition toward sustainability [19].

Plants maintain the environmental equilibrium and stabilize the ecosystem. More than 500,000 species are essential for their uses in medicine, food, fuel and clothing [20]. Plants are significant for agriculture; for a long time, plant-based agriculture and forestry have negatively impacted others [21]. Today, traditional commodities and new value-added markets can be simultaneously served; what is more, there is significant biosource and bioprocess innovation for biobased industrial products.

Plant-based alternatives are no longer a novelty on the shelves of European supermarkets and shops. The European plant-based food industry (meat, milk, yogurt, cheese, ice cream and fish) increased by 49% (2018–2020), with total sales of EUR 3.6 billion. This significant increase in plant-based food sales will allow the food industry to pursue plant-based options further. Alternative terrestrial protein sources are expected to promote the regeneration of the environment, the feasibility of processing and consumer confidence and acceptance, and, as an effect, the development of next-generation economical, environmentally friendly, nutritious and intelligent protein foods [22]. Although the plant-based food market is still just beginning, there are many startups, some of which are very promising [23].

While plant-based innovation can be easily associated with food [24,25], there are also other fields in which such innovations can be implemented, such as pharmacy [26,27,28,29,30], energy [31], textiles [32] and biodegradable-packaging production [33]. Considering all of the above, it is worth studying the problem of plant-based innovation.

Regarding sustainability, plant-based meat and dairy alternatives are potential substitutes for animal-sourced foods and allow transition to more sustainable diets [34,35]. A sustainable diet has the following attributes: it protects and respects biodiversity and ecosystems, has a low carbon footprint, is nutritionally adequate, healthy and safe, and acceptable from both cultural and economic points of view [36]. Plant-based meat production uses up to 99% less land, 90% less greenhouse gas, 99% less water and causes up to 91% less aquatic nutrient pollution than conventional meat production [37]. Innovations related to precision processing of raw materials in the plant-based food sector [38] and secondary processing of medicinal plants have also contributed to economic growth and environmental sustainability [39].

One of the scholar’s roles is to provide an overview, analysis and synthesis of findings obtained in previous research [40] to advance investigation. So far, more literature reviews have been published on general topics, such as the conceptualization of plant-based diets [41,42] and the benefits of plant-based food/diet and market trends in this area (e.g., [41,43,44,45,46,47]), than studies strictly devoted to plant-based innovation. However, in some literature reviews, the link between plants and innovation can be found. Their authors have focused on narrow research topics, such as cultured meat [48], plant-based meat alternatives [48,49], specific plants (e.g., carrot [27]), aloe vera [50], bamboo [32]) and their uses as medicines [28,51,52] or as sources of specific compounds for drugs, and innovation in plant cultivation through genetic improvement [53], for which sophisticated techniques [54,55] are needed. They have also discussed the latest applications of food metabolism, from farm to farm [56], ethical aspects of the interaction between humans and animals, from the perspective of animal welfare and rights, meat made from cells and plants [57], and consumer behavior [47]. However, the mentioned authors did not specify the types of reviews they performed or the methodologies they used. Only Aschemann-Witzel et al. [47] clearly described their sample, which was limited to scientific peer-reviewed articles published over the past five years; however, they analyzed consumer behavior in the context of sustainability, not plant-based innovation. Furthermore, the authors of the studies mentioned above did not provided bibliometric analyses. Bibliometrics is a statistical method that can be used to quantitatively analyze research papers dealing with a particular topic mathematically. It also gives the opportunity to assess research quality, analyze the main research areas and predict the direction of future research [58].

This study aims to fill the literature-review gap by presenting an analysis of publications devoted to the issue of plant-based innovation in a broader sense. To our knowledge, this is the first study to assess plant-based innovation not only in the context of the food industry and consumer behavior but also from the perspective of other possible plant-based innovations in the transition to sustainability.

In particular, an attempt was made to answer the following research questions:

RQ1: Which are the most influential countries, institutions, authors, journals and articles in the field of plant-based innovation?

RQ2: What are the main research areas in plant-based innovation?

RQ 3. What are the directions for future research?

Webster and Watson [59] stated that an effective review creates a firm foundation for advancing knowledge and facilitating theory development. This study contributes to the development of academic knowledge by introducing a holistic approach to plant-based innovation, analyzing the material in quantitative and qualitative ways, and synthesizing previous studies. The added value of this study is that it also sets directions for further research and provides valuable materials to practitioners interested in plant-based innovation.

The rest of the article is organized as follows: The second section introduces the methodology and methods used in this study. The third section shows the results after the bibliometric, scientometric and in-depth content analyses of 124 articles. Then, a discussion of both the practical and theoretical implications is provided. Finally, the significant findings and limitations of the study are summarized and conclusions are formulated.

## 2. Materials and Methods

The methodology for a bibliometric-based literature review was described by Khan [60] and Moher et al. [61] and is presented in Table 1. The PRISMA (Preferred Reporting Items for Systematic Reviews and Meta-Analysis) screening procedure was followed, as suggested by Moher et al. [61].

The authors performed a descriptive analysis to identify the distribution of articles by year of publication, the journals, the authors’ contributions and the contributions of different universities and other countries/regions. After the descriptive analysis, we performed a scientometric analysis of the 124-article sample using the VOSviewer ver. 1.6.18. software (Centre for Science and Technology Studies, Leiden University, Leiden, The Netherlands) for the construction of the scientific map. The methodology and assumptions of such an analysis were described by Eck and Waltman [62].

Finally, an in-depth content analysis was performed to identify and characterize the main research areas. The methodological approach to the content analysis was described in detail by Duriau [63] and Seuring et al. [64]. The authors used grounded theory: although the research questions were formulated at the beginning of the process, we considered possible categories of research areas that could have emerged at subsequent stages, for example, keyword network analysis. The authors read all articles and highlighted findings and insights in the text that seemed relevant to RQ2 [65]. To avoid personal bias, all authors contributed directly, working independently, carefully examining each document, comparing their results and constituting the categories of research areas; this increased the validity of the work [66].

## 3. Results

### 3.1. Descriptive and Scientometric Analysis

The analysis of the articles published from 1995 to 2022 (January) revealed that plant-based innovation is gradually gaining researchers’ attention worldwide (Figure S1). From 1995 to 2014, a small number of publications appeared, followed by an increasing publication trend.

Eighteen journals have published two articles or more (Table S1), contributing 33.8% of the articles published within the studied domain. Among them, *Trends in Food Science & Technology* published seven manuscripts, the most significant number of articles from 1995 to 2022, and it is the most active journal in the analyzed research domain. The high number of journals active in publishing articles on plant-based innovation suggests that different points of view are presented to diverse audiences; however, the titles of the most productive journals indicate that plant-based innovation primarily occurs within food science and is highly related to sustainability. At the top of this list is *Public Health Nutrition*, with an article published in 2011 being the most cited (746).

There were four universities with affiliates who had authored five or more of the articles identified: the University of Pittsburgh, USA (8); Chulalongkorn University, Thailand (7); the University of Cape Town, South Africa (6); and the University of São Paulo, Brazil (5). There was no university found to have a strong representation of studies within plant-based innovation.

Authors from the USA published 56 articles on plant-based innovation, Italian authors 28 articles, German authors 24, Brazilian authors 23, Netherlandish authors 20 and Spanish authors 19 articles (Figure S2); however, the distribution of articles by countries/regions was different from the distribution of the countries/regions of the authors.

Aschemann-Witzel, J.; Hekkert, M.; Kalfagianni, A.; Negro, S.; and Tziva, M. published three articles and were the most productive authors, with the highest individual contributions. Twenty-nine other authors published two articles. The rest (448) published only one paper.

The contributions of the articles to the studied subjects were analyzed with respect to their total citations and normalized total citations (Table S2). The article with the highest number of citations (746) was Bach-Faig (2011), followed by Thomford (2018).

Keywords provided by the authors that occurred more than three times in the analyzed database were taken into account in the final analysis, using fractional counting in VOSViewer. The exact meaning was semantically combined, for example, “plant-based protein” and “plant-based proteins”, “human” and humans”, “animal” and “animals”, “transgenic plant” and “transgenic plants”. Terms such as “article”, “review” and “priority journal” were excluded. As a result, 100 of the 1665 keywords met the criteria.

The keywords displayed in Figure S3 are clustered into six groups. Keywords in the same cluster have close interrelationships; for example, “medicinal plant” is co-studied with “drag effect”.

As indicated in Figure S3A, six themes among plant-based innovation studies were found.

The red cluster includes research on functional foods and diets, phytotherapy and phytochemicals, drug mechanisms and possible effects on health. The green cluster research is focused on sustainable development, life-cycle assessment (LCA) related to food production, quality and food safety, protein transition from beef to legumes and consumer acceptance. The blue cluster represents research on consumers’ attitudes, behaviors, tastes and perceptions of plant-based diets and plant products (meat alternatives, milk, plant proteins, plant-based proteins). The yellow cluster includes studies on molecular farming and genetically modified and transgenic plants. In the pink cluster are studies on economics and innovation (patents) in biomass and food. Finally, the research in the light blue cluster is concentrated on environmental protection.

Figure S3B shows the network map of the trend topics according to the keywords used from 2015 to 2021, and the indicator shows the current publications from purple to yellow. More recent studies on consumer behavior and acceptance, food supply, cultured meat, protein, greenhouse gases and carbon footprints have been published. Circle size represents the appearance frequency as a keyword, and the distance between two circles indicates their correlation.

### 3.2. Content Analysis

Cluster keyword analysis and in-depth content analysis led to the determination of five leading research areas:

- Agricultural and environmental innovations;
- Food/feed innovations;
- Pharmaceutical/medicinal innovations;
- Innovations related to technology;
- Economic/business aspects of plant-based innovations.

#### 3.2.1. Agricultural and Environmental Innovations

Regarding agricultural innovations, the authors concentrated on applying improved plants for various purposes, e.g., remediation [67,68] and novel crops [69].

As Bleicher [67] mentioned, a better understanding of technological innovation processes in remediation could increase the use of plant-based technologies in remediation and site management. One of the innovative solutions based on incorporating plant-based surfactants into in situ chemical oxidation technology was patented and successfully implemented [70]. However, the use of novel crops in a simple manner requires a modification of habits. Frisio and Ventura [69] suggest that RNA interference (RNAi) is an innovative technique for plant improvement based on naturally occurring mechanisms. They show the great potential of RNAi-based plants and products to support modern agriculture in advancing the global sustainability of agri-food chains.

Environmental aspects were the interest of Batlle-Bayer et al. [71], who performed life-cycle assessments of 15 tapas meals eaten in a Spanish restaurant. They stated that the meals differed in terms of energy and nutrient contents. Another aspect of the studied literature relates to the environmental factors of innovation connected with using plant residues. For example, plant-based residues (wood residues) were used as biomass [72] in an innovative biorefinery system for bioethanol production. New technologies based on vegetable biomass were studied to produce sustainable energy and societal agreements about them [73].

An interesting and innovative solution is the development of a dispersed treatment system which comprises anaerobic digestion phase and unprocessed treatment phase sub-systems placed in a greenhouse. Chen and Fukushi [74] maintained that the elevated CO2 concentration could be upscaled and used by rural communities worldwide to reclaim and reuse strong wastewater, confront agricultural pollution and promote sustainable development.

Governments support energy plants that use crop residues to ensure market presence [75]. Research should focus on technological innovations that improve the net efficiency of activities and reduce the costs of raw materials. Vicente-Vicente and Piorr [76] suggested that using climate-friendly irrigation to move to more organic-vegetable consumption is an appropriate solution to increase self-sufficiency and reduce carbon footprints. National authorities should include these results in their environmental and food-security policies.

The environmental benefits of plant-based diet meals have been studied by many authors [71,77,78,79]. Changing to healthy diets with reduced meat consumption has significant potential to reduce greenhouse gas emissions in the EU. Additionally, no innovations or significant investments are required, and substantial health cost savings can be made. This statement agrees with Hardy et al. [21], who stated that benefits such as self-sufficiency in fuel transportation to more sustainable industries, revitalization of rural economies, improved balance of payments and mitigation of environmental problems could be achieved. One of the global challenges for sustainable and responsible companies is corporate social responsibility. Many companies are implementing a sustainable development approach. For example, Roquette, a family-owned group (a leader in plant-based ingredients for food, nutrition and health markets), has planned that, by 2025, a minimum of 70% of its innovation projects will meet the criteria of green chemistry. They also intended that a minimum of 80% of their innovative solutions offered to customers should meet sustainability requirements [18].

Recent meat substitutes are often sustainable, in line with consumer concerns about the impact of food safety and food production on the environment [34]. As Zhao et al. [65] stated, food sciences must be used efficiently to achieve large-scale artificial seafood production and meet the human demand for seafood. There is a need to develop a bioreactor that can be stable, reused and differentiate muscle tissue [80].

#### 3.2.2. Food/Feed Innovations

Plant-based food or feed innovation is focused mainly on developing new products, such as meat substitutes [79,81,82], cultured meat [83], plant-based drinks [84] and new food ingredients [85].

Global protein demand is increasing. To meet both consumer demand and achieve the desired eating experience, the performance of non-protein ingredients is essential for development and manufacturing [86].

Furthermore, the number of vegetable-based meat-substitute products multiplied by five (more than 429%) in a space of four years [82]. However, a wide range of nutrients and higher sodium levels underscore the importance of having nutritional guidelines that apply to the development of such products in the same way as those that apply to animal-protein products. The commercial aspects of innovation are crucial, especially those related to molecular agriculture [81]. More action is needed to make plants more competitive and attractive to the industry.

Researchers have also developed new techniques to increase plant-based foods’ nutritional and nutraceutical values. For example, Bueno et al. [84] demonstrated the usefulness of germination in soy-based food production, and Bintari et al. [85] established a yogurt partially substituted with tempeh flour. Such a new product might be considered a functional food, especially for consumers with metabolic diseases. The proposed innovative solution involves the extraction and production of ecologically sustainable plant compounds (phytocomplexes) from natural bioactive plants for incorporation as plant-based ingredients and/or dietary supplements (botanicals) [87].

The potential for food innovation and reformulation of existing recipes will contribute to multiple sustainable development goals, reduce food waste and change dietary behavior. The partial replacement of a lean beef burger with cooked lentil puree increased the dietary fiber content 60 times, the total folate content 3 times, the manganese content 5 times and the selenium content 1.6 times compared to an all-beef burger [79].

Innovation also exists in the food pyramid. Bach-Faig et al. [88] advocated recommendations for contemporary Mediterranean populations considering diet, socio-culture, the environment, health and lifestyle challenges. The primary recommendations are to ensure that main meals are plant-based; to exercise economy and prudence; take into account qualitative lifestyle factors and cultural elements, such as sociability, culinary activities, physical activity, adequate rest and balance, and frequency advice for food consumption. The authors created a new diet pyramid that considers food production, seasonality, biodiversity, and traditional, local and environmentally friendly products.

Broekema et al. [78] declared that meeting nutritional requirements and greenhouse-gas-emission targets is feasible. However, achieving varied and adequate diets given the availability of modern food products is challenging. In addition, as Zech and Schneider [77] postulated, if healthy diets were embraced and meat intake reduced from around 200 to 100 g per day per head, biofuel production could increase by more than sevenfold.

The food sector is increasingly turning to sustainability issues. However, the policy and market context are favorable only for the near future. Consumer beliefs, perceptions and understanding have to change further, especially concerning novel alternatives, including novel meat substitutes (cultured meat and highly processed plant-based meat alternatives) [47] as well as insects and novel protein sources (such as algae) [24], for the business opportunity to grow on a larger scale.

Acceptance of cultured meat by consumers is not guaranteed [83]. Further technological developments are required to continuously improve the efficiency of animal, plant and cultured-meat production. However, when promoting plant-based food, some deficiencies, such as vitamin A deficiencies, especially in young children, should be avoided due to the low bioeffectiveness of the diet [89]. The limits of alternative dairy products based on plant proteins are a relatively low protein content and sensory appeal [90,91]. The food industry, public health regulators and standardization bodies should adopt the nutrient standards of this category [92].

It should be noted that young consumers with positive views on sustainable diets, favorable attitudes toward food innovation, motivation to pursue sustainability and who are nutritionally informed are more likely to buy mixed meat–mushroom burgers. These results can help marketers, policymakers and retailers better understand the behavior of young consumers and identify strategies to promote their transition from meat-based to plant-based food [93]. With a growing consumer trend toward meat reduction and more plant-based diets, the food industry is developing meat-reduced food innovations, such as blended products which partially replace meat ingredients with plant-based ingredients. These products are generally promoted as being more sustainable than existing ones [94]. The sustainable vegetable diet is based on food science, culinary innovation and food design. It is necessary to analyze consumer preferences and willingness to pay for sustainable food produced with new agricultural technologies, accelerating the transition to a sustainable food system [95]. Growing consumer health awareness has forced the food industry to develop a new alternative similar to existing food products (e.g., reduced-meat or plant-based burgers) [96].

Plant-based and flexitarian diets are gaining in popularity, and the food supply system has responded with a wide range of convenience products; however, there is still a lack of understanding regarding consumer views [97]. Consumers expect food innovation to address sustainability signals. They want products that are sustainable, healthy and better for the welfare of animals. However, they must overcome barriers to changing their diets, such as culinary skills, taste preferences and family support [36]. The need for sustainable alternatives to animal-derived proteins has accelerated the development of plant-based innovations. Oat proteins are potential candidates as performance ingredients for tailoring taste and texture. Enzymatic modification is a valuable tool for designing plant-protein ingredients with tailored functional properties [98]. The main objective of producers is to offer consumers new attractive products, even if traditional fermentation meat-sausage aromas have not been achieved [99].

Raw plant ingredients often lack the essential flavors of umami and sweetness and require special attention to the mouthfeel. A condiment “taste rack” is a kind of generalized spice rack or taste inventory that allows most vegetables to be transformed into tasty dishes by “umamification”. It was developed to be effective in the flexitarian context [100]. Acceptance drivers, such as motives of taste and health motivations, familiarity, attitudes, food neophobia, disgust and social norms, are consistently related to the acceptance of different alternative proteins [101].

Various processes have been tested to improve plant-based protein functionality (e.g., increasing solubility and hydrolysis). However, there is a risk of adverse nutritional effects because synergies between plant and animal proteins can create nutritious and attractive foods [102].

Soy milk, an alternative to cow milk, is known for its health and nutritional benefits. New technologies and research are underway to develop soy milk with maximum nutritional quality [103]. Current trends create opportunities for incorporating plant-based proteins into milk to produce high-protein, multisource, functional foods. Plant proteins, such as pea protein, can be challenging to use in the food system due to their low solubility and unpleasant flavor. Low-temperature homogenization can be used to produce a colloidal dispersion with high stability. Plant-based proteins in the dairy industry will help drive product innovation and meet current processing conditions and consumer demands [104].

#### 3.2.3. Pharmaceutical/Medicinal Innovations

Articles belonging to the medicinal/pharmaceutical research domain were primarily focused on new plant-origin ingredients to be used in pharmacy [105], medicine [106,107,108,109,110,111] and cosmetics [112].

Purifying a plant-based allergen in a green alga opened the door to specific immunotherapies against allergies [106], the production of which is both cost-efficient and straightforward. This solution might replace unpleasant injections with oral administrations.

Some innovations in oral products containing plant-derived compounds with antifungal activity (anti-Candida) have recently been developed [108]. They are also desirable innovations in the pharmacodynamics of antipsychotic treatment [111], as well as plant-based medicines for animals (veterinary phytomedicines) [113]. An increased number of applications involving cosmetic-containing plant components related to anti-aging and whitening skin treatments can be observed [114].

Genetically modified plants and transgenic seeds could be used to develop and produce new pharmaceutical vaccines or products [109]. For example, carrot (*Daucus carota* L.) was used to produce biopharmaceuticals (vaccines and enzymes) for human use [27].

Therapeutic plant-based compounds have been widely studied in preclinical trials [29] of, e.g., anticancer and antimalarial therapies [110].

#### 3.2.4. Technology-Related Innovations

The articles identified as presenting technological innovations focused mainly on special techniques to improve production, or on finding new solutions with or applications for plant-based materials. For example, electronic nose (enose) instruments, developed from various aroma sensor technologies, are used profitably to improve plant-based products’ characteristics, quality, consistency and uniformity [54]. Hao [115] suggests that genetic breeding research will enter a new stage with subgenetics and the continuous innovation of molecular-marker technology. Alternative plant-based fibers, such as plant-based lycra fiber [116] or bamboo, are the most recent innovations in the textile industry; they could replace petrochemical-based synthetic fibers [32]. Innovations in the textile industry also include a local, natural, plant-based indigo dye to produce silk fabrics [117].

Hughes [118] introduced biodegradable liquids (gear oils and hydraulic fluids from plant-based technology) for numerous industrial gear applications.

The growing environmental concern about plastic-packaging disposal has led to the innovation of biodegradable biopolymers [119], such as microfibrillated cellulose [105] and natural pigments [120]. Biodegradable packaging is economical, safe, nontoxic and sensitive, and natural pigments can act as quality indicators in packaging systems. Furthermore, these packaging films can be optimized, commercialized and used as active and intelligent packaging for the visual quality evaluation of fresh food products [33]. Another innovative process is to produce packaging from biological waste and fungi (packaging made of seaweed is 100% biodegradable and edible) [120].

It is worth noting that innovations are generated mainly in universities; their revenue streams come from licensing plant-based innovations from fees, royalties and a combination of the two mechanisms under exclusive and nonexclusive contracts [121].

#### 3.2.5. Economic/Business Aspects of Plant-Based Innovations

The authors of articles classified as “economic/business aspects of plant-based innovations” discussed consumer preferences, reactions and market transitions.

Regarding consumer preferences, Fischer et al. [81] researched consumers familiar with or interested in plant-based food. The authors showed that specifying the origin of a protein ingredient engenders a more positive view of the product. As a consequence of this investigation, potato protein was found to be especially advantageous with respect to an abundance of subjective quality aspects. This research showed that food producers could increase the positive perception of a product among consumers by indicating and highlighting the exact protein type on the ingredient list.

In turn, McCarthy et al. [122] showed which attributes of plant-based beverages are most appealing or essential to consumers and why they are crucial in private life (balanced diet and healthy lifestyle). The most critical attributes for plant-based beverages were the sugar level, the source of the plant and package size. The most popular plant source was almond milk, and the four-pint packaging was the preferred package size. The respondents who only drank non-dairy vegetal-based alternatives were motivated by the fact that vegetal-based drinks contribute to the consumption of fewer animal products as well as by views regarding animal mistreatment and the perception that such drinks have a less harmful effect on the environment compared to animal milk.

Graça et al. [123] investigated how consumers might respond to different policies, why they react as they do and how to raise public support for policies. Participants characterized as having an environmental ideology had a more positive attitude towards meat-curtailment policies. Compared to others, participants who supported human-primacy beliefs and were more closely tied to meat consumption were less receptive to these policies. Despite these associations, reading about legal approval raised participants’ support for meat-curtailment policies despite individual differences in philosophy and consumption habits. This suggests that communicating statutory innovations on the subject may support policies that promote increased vegetal-based diets.

As Aschemann-Witzel and Peschel et al. [47] suggested, the food sector needs to become more resource-efficient due to the impending sustainability challenge. This involves moving towards an integrated approach whereby by-products from manufacturing side streams are reintroduced into the food chain.

In an experimental qualitative and quantitative study involving 495 consumers in Denmark, Aschemann-Witzel and Peschel [124] suggested that analogous differences influence attitudes toward such novel ingredients. Communication could improve consumers’ less favorable attitudes toward vegetal-based products containing protein.

According to Broad [125], meat is no longer connected to animal farming; it is a combination of tastes and textures that can be reconstructed using food science and biotechnology. Regarding the market for alternative meat products, innovation, capital investment, behavioral economics and marketing insight are the main factors contributing to the realization of the “post-animal bioeconomy.”

Stakeholders involved in the supply chain for food products (processors, producers and distributors) were critical of new vegetal-based foods, highlighting problems with taste, processing technology and cost. These results contrast with the views of policymakers, environmental NGOs, researchers and consumers who see these new products as healthier, more sustainable and profitable [126]. Changing practices is a significant issue for sustainability. Therefore, some authors have focused on more general problems related to the market transition [127], while others have focused, e.g., on specific industries (e.g., [128]) or products (e.g., [129,130]).

Tziva et al. [127] showed how improving cognitive and normative legitimacy could increase the demand for sustainable products. The engine starts with collectives of actors and/or organizations, including NGOs and independent organizations, who accept an emerging standard and support the advance of products with distinct characteristics because they comply with this standard. When government actors participate, they contribute financially through subsidy programs to improve product performance, which leads to the establishment of entrepreneurial and awareness-development projects. At the same time, increasing the acceptance of products positively influences market growth. It should be noted that this author also indicated directions for future research, such as identifying the relationship between emerging norms (e.g., political context) and the legitimation process of technology-innovation systems and exploring the role of a broad range of actors.

Gravely and Fraser [129] focused on the role of supermarkets in the market-transition process. They stated that discussions about how best to increase consumption by the Canadian society of alternative protein products would greatly benefit from a better understanding of supermarkets’ role in this progression. They observed a change in supermarket behavior; for example, more vegetal-based proteins are placed in the busy “fresh” sections and vegetal-based ersatz meat and dairy products are placed on the same shelves as those products they try to mimic.

Policy interventions are needed to reduce the negative results of conventional agricultural and forest biofuels [131]. For example, the actors in the plant milk market will also be reconfigured due to cultural factors [130]. The transformation of the traditional farming industry has made it more sustainable thanks to innovations in feed (for example, salmon farming) [128]. Additionally, a transition to haute cuisine has been observed, and local food production and consumption is expected to continue to be a significant trend, enhanced by insects, vegetal-based proteins and complex nonalcoholic food pairings [132].

As Tambo [133] showed, the transition to sustainability can be stimulated by grant programs. Farmers have valuable ethnobotanical knowledge and are eager experimenters. They can produce notable and locally adapted innovations, indirectly contributing to attempts to sustain agricultural intensification and make agriculturalists aware of climate change. However, awareness of farmer innovations needs to be increased among the relevant stakeholders. One of the solutions could be microcredit, a form of government intervention that has been verified to significantly impact the capacity of farmers in Northwest Vietnam [134].

Cusworth et al. [135] suggested that macroeconomic changes and political transitions combined with the evolution of agricultural attitudes will increase the appeal of crop diversification and legume farming. These changes (growing markets for plant proteins and focusing on public goods subsidies, soil-health concerns and long-term profits over annual production) create space for microinnovation for actors in the private, public and civil society sectors, disrupting the status quo of microbial crop rotation with minimum diversity.

It is necessary to emphasize the development of processing technologies and supply chains, which could improve the socio-economic performance of legume-based foods. This development would increase the supply of sustainable protein-rich foods and make them more economically attractive. Policies should be oriented at different stages of the food value chain to maximize the development of innovative plant-based foods [136]

There is a link between trade and investment, the spread of unhealthy food products, efforts to impede nutrition labeling and increased concentrations of ultra-processed food and beverage product companies. The role of trade and investment in reducing animal-sourced products in human diets is becoming apparent. This role may include challenging measures that restrict the use of terms such as “milk” and “burger” in reference to plant-based alternatives and the promotion of plant-based foods through non-tariff barriers and targeted efforts at regulatory harmonization. Trade disputes can be a forum for battles around state discrepancies regarding the safety and acceptability of technological innovations in food supply [137].

The rules for the naming and labeling of plant-based products have provoked controversy. From a commercial point of view, claiming health is as tricky as the process of developing new foods. EU food laws must ensure food safety and consumer rights while applying principles of nondiscrimination and proportionality [138].

A firm’s connections to its ecosystem influence its ability to innovate. Plant-based-protein-firm networks were found to have a greater innovation orientation than the established food-producer network, particularly among industry and civic-association intermediaries, the government and other agricultural companies [139]. Associations between various types of organizations can facilitate transitions by promoting the adoption of potentially beneficial innovations and sustainable consumption [140].

Food producers, incumbents and new entrants begin at the early stages of planning direction and goals and then change and experiment to adapt to the business environment. The company moves from experiments and learning activities to innovative implementations that drive institutional changes toward sustainability [141].

Internet and communication technologies, blockchains in the food supply chain and other Industry 4.0 applications and new kinds of food products (e.g., lab-grown meat, plant-based meat alternatives, and valorization of a vast range of bioresources) are the most promising innovations. Furthermore, social marketing must understand attitudes, perceptions and obstacles to changing consumer behavior and the agri-food industry [142].

Sebo [143] explored the moral, conceptual, social, political, economic and technical challenges that stand in the way of the widespread adoption of plant-based meat and cultured meat as alternatives. Companies and political leaders can be convinced that plant-based meat and meat cultivation are opportunities rather than threats.

## 4. Discussion

As indicated in the Introduction, previous literature reviews on similar but more general or narrower topics have not provided bibliometric analyses of the research field. This review shows that the total number of articles on plant-based innovation has increased with respect to the first research question. In turn, the high number of journals that actively publish articles on plant-based innovation suggests that different points of view are being presented to diverse audiences. Although the most productive journal specializes in food science (*Trends in Food Science & Technology*), the one with the most significant impact on the studied field is dedicated to the drug industry (*Public Health Nutrition*). Plant-based innovation is also a popular topic in journals devoted to sustainable development. The most influential countries with the highest contributions to knowledge are the United States, Italy, Germany, Brazil, the Netherlands and Spain.

Four universities have affiliates who authored five or more of the articles analyzed here: the University of Pittsburgh, USA; Chulalongkorn University, Thailand; the University of Cape Town, South Africa; and the University of São Paulo, Brazil. The most productive authors are Aschemann-Witzel, J.; Hekkert, M.; Kalfagianni, A.; Negro, S.; and Tziva, M. *Trends in Food Science & Technology* published seven manuscripts, the maximum number of articles during 1995–2022.

As for the second research question, five leading research areas related to plant-based innovations were identified: (1) agricultural/environmental innovations related to genome engineering in crop improvement and applying improved plants for remediation processes and plant-residue utilization; (2) food or feed plant-based innovations focusing mainly on new products, such as meat substitutes, new food ingredients, the nutritional values of new products and the environmental benefits of plant-based diet meals; (3) innovations belonging to the medical/pharmaceutical research domain, mainly focused on new plant-origin ingredients to be used in pharmaceuticals, medicine and cosmetics; (4) technology-related innovations focused mainly on special techniques for improved production, new solutions and the application of plant-based materials; and (5) economic/business aspects of plant-based innovations studied in relation to consumer preferences and reactions, as well as market transitions.

**Figure 1:**
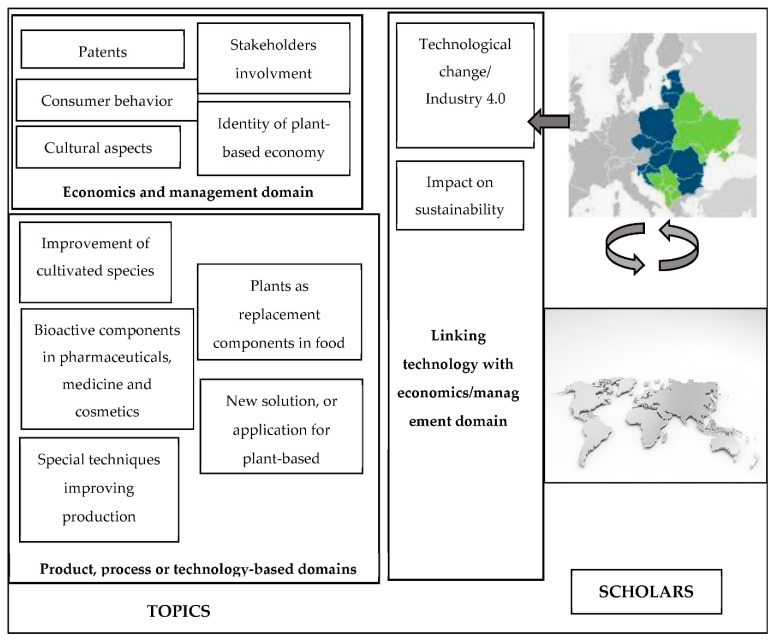
Framework for future research. The left arrow: research conducted in different countries is needed. The ringed arrows: cooperation between academics from different countries is needed.

The above presents a holistic view of the “plant-based innovation” research field and enriches the previously published literature reviews that focused mainly on plant-based foods/diets or trends in the food market (e.g., [41,43,44,45,46,47]) and medicine [28,51,52]. The third research question is related to the presentation of future research avenues (Figure 1). Some directions for future research come directly from the bibliometric and content analyses of articles on plant-based innovation. The authors identified the others independently, reflecting the authors’ original contributions to the analyzed research field.

This review shows that keywords such as “sustainability”, “food”, “nutrition” and “meat” have gained more importance in recent years, highlighting the value of the application in food/feed. Not only are there numerous research articles related to the food system, there are also—as presented in the Introduction—many literature reviews on this topic. Promising research avenues, such as technological changes and patents, might be less popular areas (being less popular keywords). There is much space for development for scientists from countries other than the most productive ones (e.g., Central and Eastern Europe). Although no university with a strong representation of studies on plant-based innovation was identified, this document indicates the most productive partners for starting cooperation.

Researchers have noted that future work is needed to improve cultivated species, for example, halophytes [144], and develop the use of different fiber species (flax, banana fiber, jute, wood-based textiles and many others) [145]. There is also future potential for the development of biomedical applications using nanostructures [50] and novel allergy-treatment concepts, such as oral administration of allergen-containing algae for therapy [106]. The commercial demand for herb-containing cosmetics has increased, so it is necessary to research bioactive components [114]. However, as suggested by Rodrigo da Silva [146], there is a growing demand for solutions involving processes that lead to sustainable development, which is becoming a challenge for science. It has also been suggested that processes and methodologies should be designed in such a way as to eliminate or reduce the use of hazardous and toxic chemicals at every production stage, not only in industry but also in the laboratory.

In addition, healthy meat replacements with clean labels are needed [47], as well as improvement in product perception [147]. However, Frehner et al. [148] noted that the reduction in the number of animal products in the diet would have to be compensated by increased or especially varied plant-based foods so that nutritional requirements are still met. Developing a land-use model that considers both foods of plant and animal origin while considering resource efficiency and flows between different production systems is necessary. This finding is in line with that of Aschemann-Witzel et al. [47], who emphasized in their literature review that consumers’ interests in health and clean labeling should be met.

As pointed out by Coucke et al. [149], consumer attitudes and opinions are based on assumptions and expectations. Shopping and trying out products is a necessary first step in the consumer’s acceptance of new and unfamiliar products. Changing eating behavior and food consumption so that they become more sustainable requires not only direct influence and encouragement but also changes to legislation [150]. In this context, it is crucial to study actual consumer behavior, examine how consumers adopt new products and use them in various practices [130], and evaluate why consumers do not regularly commit to a meat-substitute diet [151].

Moreover, consumer education should focus on trust building and nutrition [122], which He et al. [49] emphasized in their literature review on plant-based meat. It is also essential to stimulate the consumption of new plant-based, rich-in-protein foods [152]. What is more, transitioning to sustainable diets should be based on local, plant-based, unprocessed food, with limited food waste [153].

It is postulated that different stakeholders, such as government, society and academia, should be involved in the transition to a sustainable food system [154]. Policymakers and advisory associations should identify acceptable changes and guide the necessary dietary changes. Changes should focus not only on various patterns of food consumption but also on food production [78]. As suggested by Leialohilani and de Boer [155], the regulatory framework of the European Union positively affects innovation, providing legal clarity and ensuring a high level of food safety for consumers of dairy products, yet at the same time negatively affects innovation, for example, by failing to provide a legal definition of vegan food and with its narrow definitions of “milk” and “milk products”.

Several aspects that must be studied are the functionality of nutrients in products, regulatory limitations and consumer demand [156]. Additionally, it is necessary to evaluate the partial or complete substitution of salt with natural preservatives, such as herbs and spices [157,158]. Further aspects, such as price, taste, texture, product reformulation, fortification, supplementation [78] and flavor [122], must also be studied. Moreover, the change in diet may cause problems with respect to the stability and viability of probiotics in new food environments [159].

The production and sale of food affects the environment primarily through the type of energy source used, wastewater treatment, packaging and logistics [158]. Plant-based food is considered environmentally friendly and better for animal welfare than conventional analogs. The shift to a sustainable plant-based diet might significantly reduce total GHG emissions, land use, water consumption and energy use [160]. This finding corroborates studies that revealed that replacing animal-origin foods with plant-based foods might reduce food-related greenhouse gas emissions. Moreover, this might help achieve the 13th Sustainable Development Goal (Climate Action) and the Paris Agreement commitments [161]. It is worth mentioning that plant-based animal-meat analogues do not contribute to growing global health risks, such as antibiotic resistance and pandemic risk [162].

However, as pointed out Grossman and McClements [158], comparing the values of environmental indicators for plant-based analogs and animal-origin foods may be challenging due to the lack of LCAs for plant-based analogs. The main reason for this is still the early stage of technology-readiness and the lack of producers. As raw-material usage has the greatest impact on the environmental sustainability of products, such comparisons could be made based on environmental indicators of the raw materials used.

Regarding environmental indicators, it is crucial to explore the sustainability implications of incorporating various plant-based foods into beef burgers [79]. Developing strategies for energy efficiency in restaurants, innovation in menus, reducing food waste and ensuring the sustainability of eating patterns are also essential [71]. In restaurants, innovation occurs predominantly in preparation and on the plate. Restaurant business models are virtually identical. Few try to escape institutional norms and attempt to develop their business model [132].

It is also suggested to explore whether initiatives that could be included in more high-cost/direct divisions will produce moderative effects [129]. According to Ehrenfeld and Kropfhausser [13], there is still no clear concept of a plant-based bioeconomic industry, especially regarding the importance of plant-based resources and production technologies. Combining design and design thinking provides opportunities to develop new products [162].

Such additional detailed topics, which have not been presented in papers assigned to the leading research areas, are also worth exploring: (1) in relation to agriculture/the environment, the improvement of cultivated species and development of their uses; (2) plant-based food or feed innovations to introduce new natural preservatives, with more focus on sustainable production processes; (3) pharmaceutical/medical innovations, such as biomedical applications in the form of nanostructures and novel allergy treatments; (4) technology-related innovations and Industry 4.0 in plant-based innovation; (5) economic/business aspects of plant-based innovations, including national cultural factors and the creation/absorption of plant-based innovations. Since innovation often occurs at the interfaces between various fields of knowledge, the authors of this study encourage researchers to link and develop findings from articles assigned to different research areas (especially in terms of sustainability and technology, as indicated in Figure 1).

Practitioners can use the information presented in this study to create plant-based innovations in different businesses (e.g., the food industry, medicine). The information is intended to make researchers aware of the advancements in the field. Moreover, this study also provides bibliometric data. Thanks to these data, researchers may easily find journals that offer a broader scope of information on industry-specific innovations.

Based on the findings presented, policymakers and funding agencies can create and implement policies and instruments to further develop plant-based innovations. Since this study emphasizes the need for cooperation between companies and universities, policies that serve to increase this cooperation are required. As far as financial instruments are concerned, the findings presented may be used to make decisions related to funding allocations.

## 5. Conclusions and Limitations

Due to the methodological approach to a literature review and a combination of different analysis techniques, this study presents a scientific map of the holistic “plant-based innovation” research field, extending previously published reviews on the fragmented use of plants in different industries. We have provided a literature review that identifies leading research areas related to plant-based innovations in agriculture and environmental science, including food/feed, medicine/pharmacy, technology and economy/business. Our review can support various stakeholders in understanding consumer behavior and trigger more research in identified areas and the development of public and organizational policies.

However, this study has some limitations. For example, the time period selected from 1995 to 2021 might not have allowed the coverage of other, related research. The database search was constructed based on selected keywords; other related articles would have been found if there had been different associated keywords used. Moreover, non-English manuscripts were not considered in the analysis, such that potentially relevant papers published in other languages might have been excluded. In addition, this article did not analyze conference reports, books or book chapters. This review was based on the Scopus and the Web of Science databases. Future analyses may also cover other documents indexed in these and other databases, e.g., Dimensions.

The content analysis and the identification of the scientific area were also limited by the authors’ subjectivities; other researchers could have classified the analyzed articles differently. Although based on an in-depth content analysis, consideration of future research opportunities may also involve a degree of creativity and thus have a subjective element. Researchers with different backgrounds and knowledge links might have produced other suggestions.

## Figures and Tables

**Table 1:** The methodology of the literature review.

Steps	Criterion	Effect	
Parameters	Keywords: Time horizon: Database:	((‘plant-based’ or ‘plant-based’) AND innovation) 1995–2022 (including all articles published up to 31 January 2022) Scopus and ISI Web of Knowledge	
Identification	Records identified through database searching	Scopus: *n* = 189 ISI WOK: *n* = 124	Sum of records to be screened: *n* = 313
Screening	Exclusion: 1. Unpublished articles, books, book chapters, notes, tutorials, conference papers, short surveys, no abstract, errata 2. Not within the time frame 1995–2020 3. Language other than English	Records excluded Scopus: *n* = 66 ISI WOK: *n* = 23	Records screened SCOPUS: *n* = 122 (86 art., 36 rev.) ISI WOK: *n* = 101 (66 art., 35 rev.)
	Duplicated records were screened for duplicates *n* = 223	Records excluded *n* = 83	No duplicates *n* = 140
Eligibility	Full-text articles excluded (*n* = 16)	Exclusion reasons (not related to the topic) *n* = 16	Full-text articles were assessed for eligibility *n* = 124
Content analysis of the findings of the bibliometric analysis (meta-literature review)	Studies included in a qualitative synthesis	VOSviewer analysis	*n* = 124
	Studies included in the quantitative synthesis (meta-analysis)	Content analysis	Research papers *n* = 86 Review papers *n* = 38
Findings and contributions	1. Identification of research domains 2. Identification of the most influential aspects: countries, institutions, authors, journals, articles and topics 3. Identification of future research trends and limitations		

## Data Availability

The datasets generated for this study are available on request to the corresponding author.

## Supplementary Materials

The following supporting information can be downloaded at: https://www.mdpi.com/article/10.3390/foods11193137/s1, Figure S1: All published articles from 1995 to 2022 (up to January); Figure S2: Distribution of published articles by countries/regions (a minimum of three publications); Figure S3: Bibliometric analysis of themes. (A) Distribution of the themes. (B) Network map of the trend topics according to the keywords used from 2015 to 2021; Table S1: The impacts of top journals: summary; Table S2: Articles with high scores related to citations.
