# Supplementary material for: Plant-Based Innovations for the Transition to Sustainability: A Bibliometric and in-Depth Content Analysis
Source: Foods. 2022 Oct 9;11(19):3137. doi: 10.3390/foods11193137 (PMC9561981; doi:10.3390/foods11193137)
Supplement: Supplementary file 1 [file foods-11-03137-s001.zip › foods-1882473-supplementary.pdf]

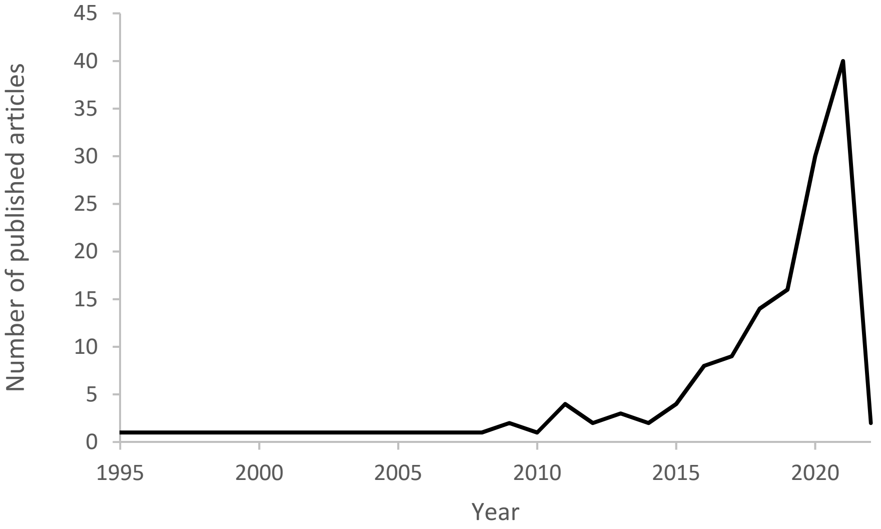

Figure S1. All published articles from 1995 to 2022 (up to January).

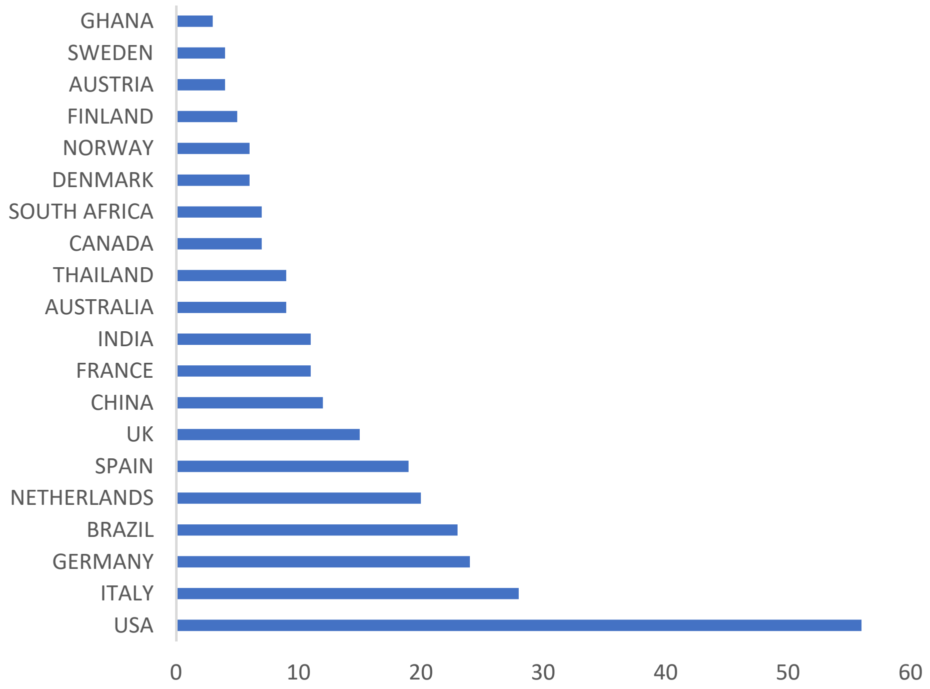

Figure S2. Distribution of published articles by countries/regions (a minimum of three publications);

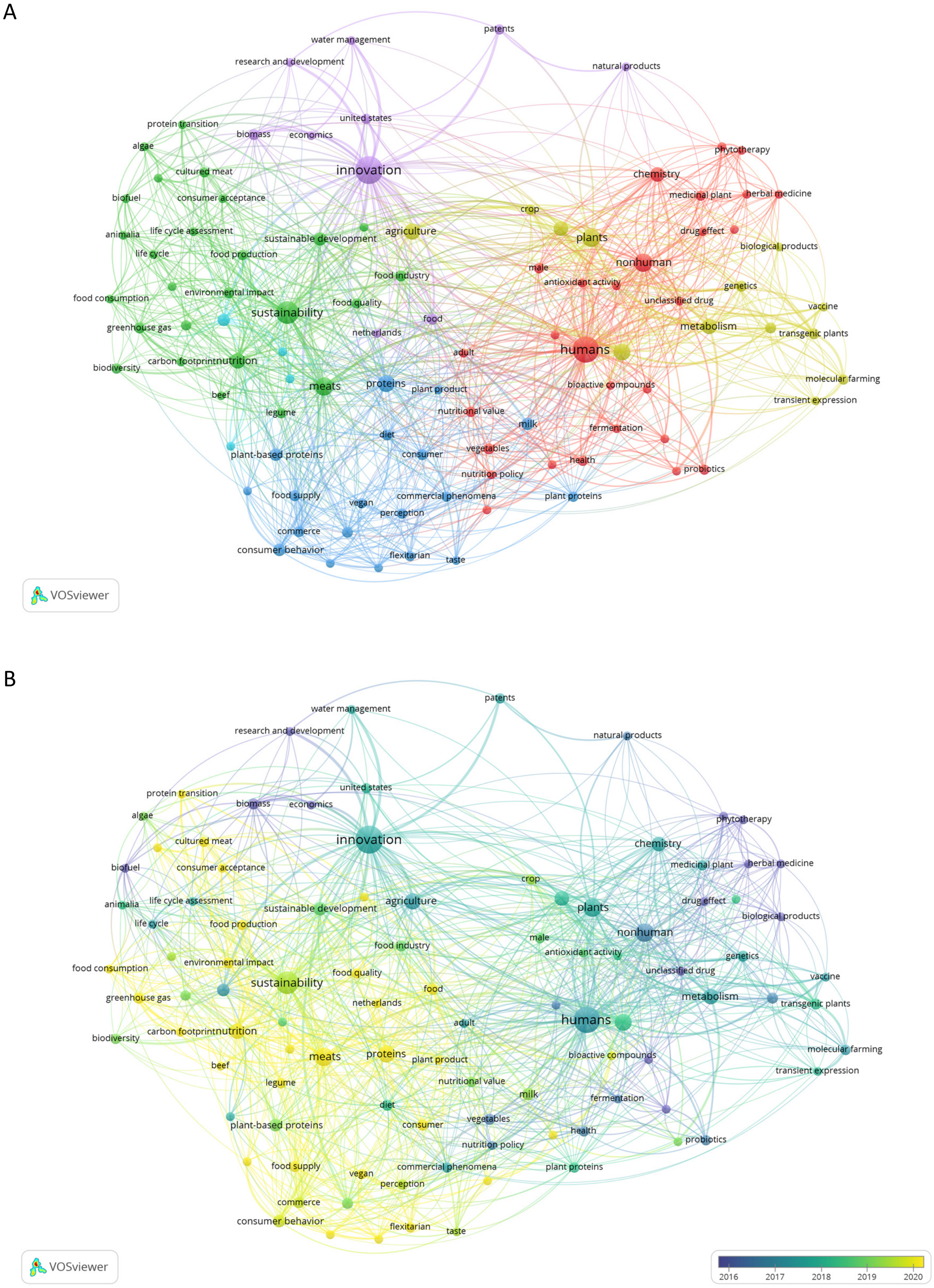

Figure S3. Bibliometric analysis of themes. (**A**) Distribution of the themes. (**B**) Network map of the trend topics according to the keywords used from 2015 to 2021.

Table S1. The impacts of top journals: summary

Source	Documents	Citations	Average publication year
Trends in Food Science & Technology	7	227	2020
Sustainability	6	46	2019
Appetite	3	36	2018
Environmental Innovation and Societal Transitions	3	97	2019
Food Quality and Preference	3	40	2019
Foods	3	16	2020
Applied Energy	2	37	2014
Critical Reviews in Food Science And Nutrition	2	119	2012
Current Opinion in Biotechnology	2	25	2020
Environmental Communication-a Journal of Nature And Culture	2	20	2020
Frontiers in Plant Science	2	21	2018
International Journal of Food Design	2	4	2020
Journal of Cleaner Production	2	5	2020
Journal of Dairy Science	2	72	2017
Journal of Environmental Management	2	14	2016
Meat Science	2	1	2021
Molecular Biotechnology	2	44	2015
Scientific Reports	2	14	2016
Public Health Nutrition	1	746	2011
International Journal of Molecular Sciences	1	301	2018
Current Pharmaceutical Design	1	81	2013
Molecular Biology and Evolution	1	78	2008

Table S2. Articles with high scores related to citations

Paper	Total Citations	Total citation per Year	Normalized total citation
Thomford N, 2018, Int J Mol Sci	301	60.2	9.892
Galanakis C, 2021, Trends Food Sci Tech	39	19.5	8.8136
Aschemann-Witzel J, 2021, Crit Rev Food Sci	37	18.5	8.3616
Onwezen M, 2021, Appetite	24	12	5.4237
Bhargava N, 2020, Trends Food Sci Tech	54	18	5.0784
McCarthy K, 2017, J Dairy Sci	72	12	4.9466
Bach-Faig A, 2011, Public Health Nutr	746	62.167	4.0
Van Der Weele C, 2019, Trends Food Sci Tech	73	18.25	3.8548
Nayak L, 2016, Fash Text	32	4.571	3.5068
Sha L, 2020, Trends Food Sci Tech	36	12	3.3856
Tziva M, 2020, Environ Innov Soc Trans	30	10	2.8213
Curtain F, 2019, Nutrients	45	11.25	2.3762
Estell M, 2021, Sustainability-Basel	10	5	2.2599
Rosales-Mendoza S, 2015, Mol Biotechnol	27	3.375	2.0769
Aschemann-Witzel J, 2019, Food Qual Prefer	36	9	1.901
Gupta S, 2012, Crit Rev Food Sci	82	7.455	1.8427
